# The effect of loteprednol suspension eye drops after corneal transplantation

**DOI:** 10.1186/s12886-021-01982-8

**Published:** 2021-05-26

**Authors:** Yingxin Chen, Xifei Wang, Minghong Gao, Ruiyao Gao, Lixin Song

**Affiliations:** 1 Department of Ophthalmology, The General Hospital of Northern Theater Command, Shenyang, P.R. China; 2 Department of Dermatology, The General Hospital of Northern Theater Command, No. 83, Wenhua Road, Shenhe District, 110840 Shenyang, P.R. China

**Keywords:** Corticosteroid-induced ocular hypertension, Corneal transplantation, 1 % prednisolone acetate eye drops, 0.5 % loteprednol suspension eye drops

## Abstract

**Background:**

To compare the effect of loteprednol suspension eye drops after corneal transplantation with the effect of prednisolone acetate eye drops.

**Methods:**

A total of 234 patients (234 eyes) who underwent penetrating keratoplasty (PKP) and lamellar keratoplasty (LKP) were retrospectively included. Patients who received 1 % prednisolone acetate eye drops were defined as 1 % prednisolone acetate eye drop group (*n* = 96), and patients who received 0.5 % loteprednol suspension eye drops were defined as 0.5 % loteprednol suspension eye drop group (*n* = 138).

**Results:**

35 cases in 1 % prednisolone acetate eye drops group and 27 cases in 0.5 % loteprednol suspension eye drops group developed corticosteroid-induced ocular hypertension, and were defined as prednisolone acetate group and loteprednol group. No significant differences were observed in the average intraocular pressure (IOP) at 1 week, 1 month, 3 months or 12 months postoperatively. There were significant differences in the average IOP between the two groups at 6 months postoperatively (*P* = 0.001). There were no significant differences in the average best corrected visual acuity (BCVA) at 1, 3 and 12 months postoperatively between two groups. The average 6-month postoperative BCVA was significantly higher in the prednisolone acetate group than the loteprednol group (*P* < 0.05). There were no significant differences in the postoperative graft rejection rates between the two groups (*P* > 0.05).

**Conclusions:**

0.5 % loteprednol suspension eye drops may be considered for long-term use after corneal transplantation.

## Background

Corneal disease is currently one of the main causes of blindness, and corneal transplantation remains the main method for visual rehabilitation when the disease affects corneal clarity [1]. Since Eduaed Zirm [2] first reported penetrating keratoplasty (PK) in the last century, corneal transplantation procedures have developed rapidly and now include lamellar keratoplasty (LK), endothelial keratoplasty (EK), Descemet stripping endothelial keratoplasty (DSEK) and Descemet membrane endothelial keratoplasty (DMEK).

After corneal transplantation, the prevention of graft rejection requires long-term use of local corticosteroids, which significantly increases the incidence of corticosteroid-induced ocular hypertension [3]. 1 % prednisolone acetate eye drops are the first choice to prevent corneal transplant rejection, but their long-term use can lead to the occurrence of steroid-induced glaucoma [4]. The various protective mechanisms of the cornea can quickly remove foreign bodies; thus, the dissolution speed of eye drops in the tears is critical. Loteprednol suspension eye drops can penetrate the cornea and travel to the intraocular tissues more efficiently than prednisolone acetate eye drops since they possess esters instead of ketones at the C20 position, which is highly lipophilic, and their affinity with glucocorticoid receptors is 4.3 times that of dexamethasone [3, 5]. Loteprednol suspension eye drops are used in the treatment of cataracts [6], corneal refractive surgery [7, 8] inflammation after corneal transplantation [4], vernal keratoconjunctivitis [9], blepharoconjunctivitis [10] and uveitis [11].

The treatment of corticosteroid-induced ocular hypertension is extremely challenging, especially after corneal transplantation, since premature discontinuation of hormonal drugs can easily lead to graft rejection and long-term use may cause corticosteroid-induced ocular hypertension [12, 13]. Therefore, in this study, we aimed to compare the effect of 0.5 % loteprednol suspension eye drops after corneal transplantation with the effect of 1 % prednisolone acetate eye drops. Compared with 1 % prednisolone acetate eye drops, 0.5 % loteprednol suspension eye drops has a weaker effect on raising intraocular pressure (IOP), similar vision loss and incidence of postoperative rejection.

## Methods

### Patients

A total of 234 patients (234 eyes) who underwent penetrating keratoplasty (PKP) and lamellar keratoplasty (LKP) at our hospital were retrospectively included in this study, including 149 males and 85 females, with an average age of 50.06 ± 15.114 years. Patients were divided into two groups depending on the eye drop treatment they received. Patients who received 1 % prednisolone acetate eye drops were defined as 1 % prednisolone acetate eye drop group (*n* = 96), and patients who received 0.5 % loteprednol suspension eye drops were defined as 0.5 % loteprednol suspension eye drop group (*n* = 138).

The inclusion criteria were patients who (1) were aged ≥ 18 years; (2) underwent PKP and LKP; (3) received 1 % prednisolone acetate eye drops after the operation; (4) used tacrolimus eye drops or cyclosporine eye drops for anti-rejection treatment; and (5) had early IOP ≤ 21 mmHg after the operation.

The exclusion criteria were patients who (1) had a previous history of glaucoma; (2) underwent multiple corneal transplantation; (3) underwent other ophthalmic surgery before or after conventional corneal transplantation; (4) had anterior corneal adhesions within one week postoperatively, and (5) had severe postoperative inflammatory reactions after ineffective conventional hormonal therapy.

The study was approved by the ethics committee of The General Hospital of Northern Theater Command (k2017-26), and all patients signed informed consent forms.

### Surgical methods

The operations were all performed by the same surgeon (Minghong Gao). Routine treatments, including eyelid margin cleaning, eyelash trimming, conjunctival sac flushing, and lacrimal duct flushing, were performed before surgery. Pilocarpine eye drops (1 drop 6 times, 5 min apart) were applied before the operation.

The PKP procedure was described in our previous study [14]. Briefly, a suitable trephine was selected to clockwise rotate into the cornea to drill the graft bed. Donor cornea was then obtained by cutting 2 mm along the limbus sclera. A trephine was used to drill the donor cornea to obtain the graft. A 10/0 suture was used to suture intermittently to bury the knot. Then, 0.05 ml of balanced saline solution (BSS) was injected into the anterior chamber. All patients were treated with tobramycin dexamethasone eye ointment.

For the LKP group, a trephine of appropriate size and diameter perpendicular to the surface of the cornea was rotated clockwise to incise the cornea. After determining the initial depth, the corneal lamina were pulled with tweezers in one hand, and the knife was held in another hand to slide the blade horizontally between the sheets of fibers to obtain a smooth plant-bed cutting surface [15]. According to the condition of the implant bed, a trephine with a diameter larger than 0.25 mm was used to drill the donor cornea, and then the corneal graft was obtained by trimming according to the ulcer lesion morphology. A 10 − 0 nylon thread suture was used. Four stitches were used intermittently to fix the implant. The line knot was buried with 10–16 intermittent stitches, and the ball conjunctiva was sutured by 10 − 0 nylon thread. The conjunctiva sac was washed again. Tobramycin and dexamethasone eye ointment were applied after eye bandage compression.

### Postoperative treatment

All patients were given 20 % mannitol injection (250 ml, twice a day) combined with methazolamide tablets (orally, 25 mg, twice a day) and sodium bicarbonate tablets (orally, 0.5 g, three times a day) to stabilize IOP. The patients were not allowed to open their eyes until 2–3 days after the operation.

All patients were given tobramycin dexamethasone ointment after the operation, pressure bandaged, and the dressing was changed daily until the eyes were opened. Then, levofloxacin eye drops (four times a day), calf blood deproteinized extract eye gel (three times a day) 3/day and tobramycin dexamethasone ointment (once a day) were given.

High-concentration hormones are required to prevent corneal transplant rejection in the first 3 months; therefore, 1 % prednisolone acetate eye drops given to patients in both groups. The hormone is gradually reduced to the maintenance level and low-concentration hormone is subsequently given. For the first 3 months after surgery, 1 % prednisolone acetate eye drops were used 4 times a day 1 month after surgery and were reduced to 3 times a day in both groups [16]. At 3 months postoperatively, 1 % prednisolone acetate eye drops were used twice a day for 1 % prednisolone acetate eye drop group, and 0.5 % loteprednol suspension eye drops were used twice a day for 0.5 % loteprednol suspension eye drop group.

### Follow-up

The patients all attended follow-up at 1 week and 1, 3, 6 and 12 months postoperatively. At each follow-up point, slit lamp microscopy was used to observe the attachment of eye grafts and graft beds. IOP, average best corrected visual acuity (BCVA) and postoperative complications were evaluated. IOP was measured using a noncontact tonometer (NCT) [17]. When BCVA was measured in Snellen, the values were converted to a logarithm of the minimum angle of resolution (logMAR) to facilitate statistical analysis. The counting of fingers, perception of hand movements and perception of light were assigned arbitrary values of 1.60, 1.90 and 2.20 logMAR, respectively. The same assumptions have been previously reported [18].

Secondary glaucoma is defined as an IOP or estimated IOP ≥ 24 mmHg and requires IOP-lowering medication intervention; when postoperative IOP is 10 mmHg higher than the preoperative IOP, or the IOP cannot be controlled by drugs, surgical treatment is required [19–22].

### Statistical analysis

Statistical analysis was performed using SPSS version 22.0 (IBM Corp.). Values are presented as the mean ± standard deviation or numbers (percentage). Normally distributed data were compared using a paired t-test or a χ^2^ test. Variables of skewed distribution were compared using a Mann-Whitney U test. *P* < 0.05 was considered to indicate a statistically significant difference.

## Results

There were no significant differences between 1 % prednisolone acetate eye drop group and 0.5 % loteprednol suspension eye drop group in the preoperative patient characteristics, including age, gender, surgical method, preoperative diagnosis and preoperative BCVA (all *P* > 0.05; Table 1).

**Table 1:** Basic information

	1 % prednisolone acetate eye drop group (*n* = 96)	0.5 % loteprednol suspension eye drop group (*n* = 138)	*P* value
Age (years)	51.03 ± 14.15	49.38 ± 15.77	0.413
Male, n (%)	55 (57.29)	94 (68.12)	0.090
Surgical method, n (%)			0.246
PKP	54 (56.25)	67 (48.55)	
LKP	42 (43.75)	71 (51.45)	
Preoperative diagnosis, n (%)			0.468
Bullous keratopathy	3 (3.13)	2 (1.45)	
Keratoconus	14 (14.58)	22 (15.94)	
Corneal degeneration	7 (7.29)	5 (3.62)	
Corneal mass	2 (2.08)	1 (0.72)	
Corneal leukoplakia	21 (21.88)	28 (20.29)	
Corneal dystrophy	8 (8.33)	4 (2.90)	
Keratitis	4 (4.17)	11 (7.97)	
Pseudopterygium	1 (1.04)	1 (0.72)	
Non-infectious corneal ulcer	3 (3.13)	4 (2.90)	
Infective corneal ulcer	27 (28.13)	53 (38.41)	
Infectious corneal ulcer perforation	6 (6.25)	7 (5.07)	
Preoperative IOP	12.41 ± 2.90	12.28 ± 3.07	0.754
Preoperative BCVA	2.04 ± 1.06	2.17 ± 1.09	0.339

*BCVA *best corrected visual acuity, *IOP *intraocular pressure

Age, preoperative IOP and preoperative BCVA were tested using a student’s t-test. The gender, surgical method and preoperative diagnosis were analyzed by χ^2^ test

### Postoperative IOP

**Fig. 1:**
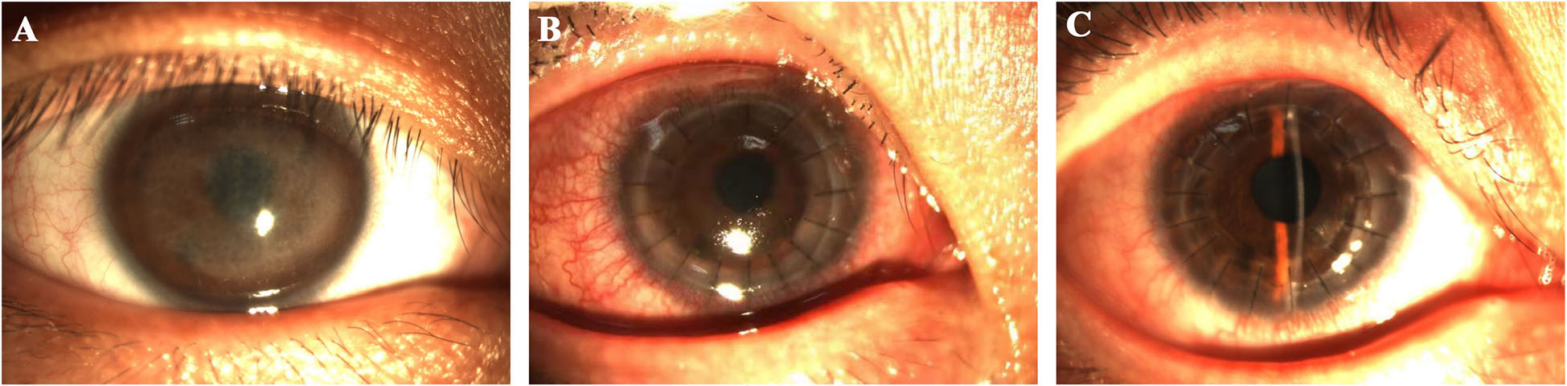
Steroid-induced glaucoma after PKP. **a**. Before operation; **b**. The IOP increased at 3 months postoperatively and the graft was slightly swollen; **c**. The IOP was normal at 6 months postoperatively and the graft was transparent

**Fig. 2:**
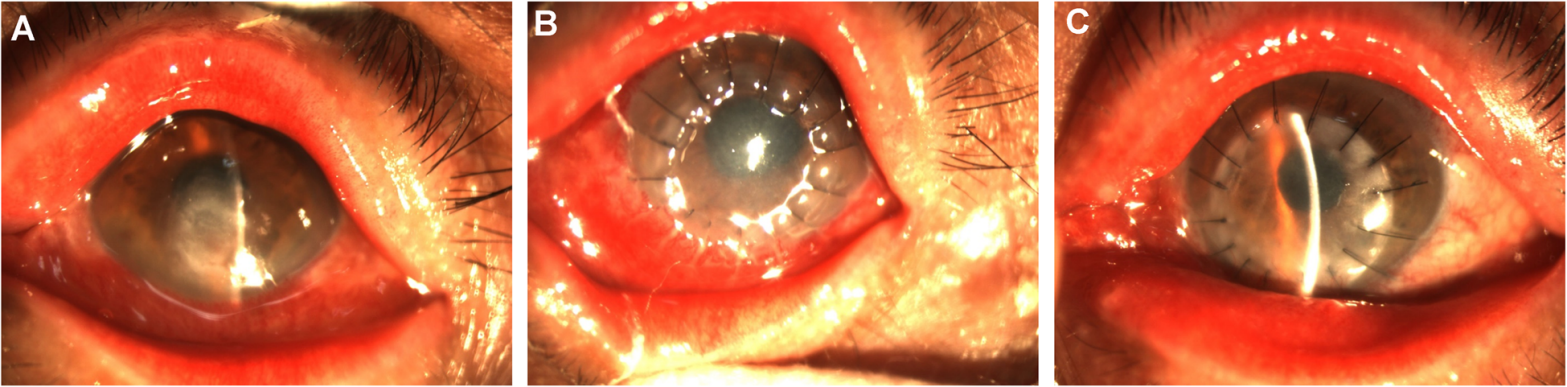
Steroid-induced glaucoma after LKP. **a**. Before operation; **b**. The IOP increased at 2 months postoperatively and the graft was slightly swollen; **c**. The IOP was normal at 3 months postoperatively

Thirty-five cases in 1 % prednisolone acetate eye drop group and 27 cases in 0.5 % loteprednol suspension eye drop group developed corticosteroid - induced ocular hypertension, which were defined as prednisolone acetate group and loteprednol group, respectively. There were no significant differences in the preoperative characteristics of prednisolone acetate group and loteprednol group, including age, gender, surgical method, and preoperative diagnosis (all *P* > 0.05; Table 2). As shown in Table 3, no significant differences were observed in the preoperative IOP and the average IOP at 1 week, 1 month, 3 months, or 12 months postoperatively. The average IOP in the loteprednol group was significantly lower than that in the prednisolone acetate group at 6 months postoperatively (*P* = 0.003). The incidence of steroid-induced glaucoma after PKP and LKP is shown in Figs. 1 and 2.

**Table 2:** Basic information

	Prednisolone acetate group (*n* = 35)	Loteprednol group (*n* = 27)	*P* value
Age (years)	53.21 ± 11.44	53.75 ± 15.37	0.874
Male, n (%)	17 (48.57)	22 (81.48)	0.973
Surgical method, n (%)			0.746
PKP	22 (62.86)	16 (59.26)	
LKP	13 (34.14)	11 (40.74)	
High risk keratoplasty	5 (14.29)	4 (14.81)	0.953
Graft bed size > 8.5mm	3 (8.57)	2 (7.41)	
Eccentric graft bed	2 (5.71)	2 (7.41)	
Preoperative diagnosis, n (%)			0.225
Leukoma	12 (34.29)	6 (22.22)	
Cornea dystrophy	4 (11.43)	2 (7.41)	
Corneal degeneration	1 (2.85)	2 (7.41)	
Keratoconus	3 (8.57)	1 (3.70)	
Infectious corneal ulcer	12 (34.29)	13 (48.15)	
Infective corneal perforation	3 (8.57)	3 (11.11)	

Age were tested using a student’s t-test. The gender, surgical method, high risk keratoplasty and preoperative diagnosis were analyzed using χ^2^ test

**Table 3:** Comparison of average IOP between the two groups

Time	Prednisolone acetate group (*n* = 35)	Loteprednol group (*n* = 27)	*t* value	*P* value
Preoperative	13.26 ± 2.97	12.26 ± 3.02	1.30	0.198
Postoperative 1 w	13.51 ± 3.22	12.96 ± 3.62	0.63	0.529
Postoperative 1 m	15.40 ± 3.77	14.81 ± 3.34	0.64	0.527
Postoperative 3 m	18.47 ± 4.87	21.18 ± 7.03	-1.22	0.229
Postoperative 6 m	21.34 ± 4.46	17.50 ± 3.88	3.12	0.003
Postoperative 12 m	17.39 ± 6.38	16.06 ± 4.50	0.82	0.413

*IOP *intraocular pressure

Data were analyzed by paired t-test

As shown in Table 4, there were significant differences in the number of patients IOP < 30 mmHg (*P* < 0.001) in the prednisolone acetate group (33 eyes, 94.29 %) compared with the loteprednol group (26 eyes, 96.30 %).

**Table 4:** Comparison of postoperative IOP between the two groups

Time, n (%)	Prednisolone acetate group (*n* = 35)	Loteprednol group (*n* = 27)	*P* value
≤ 30mmHg	33 (94.29)	26 (96.30)	0.000
> 30mmHg	2 (5.71 )	1 (3.70)	0.083

*IOP *intraocular pressure

Data were analyzed by χ^2^ test

### Postoperative diseases related to corticosteroid-induced ocular hypertension

As shown in Table 5, no significant differences were observed in leukoma, corneal dystrophy, corneal degeneration, keratoconus, infectious corneal ulcer or infective corneal perforation between the two groups after corneal transplantation (*P* > 0.05).

**Table 5:** Postoperative related diseases of corticosteroid-induced ocular hypertension

Time	Prednisolone acetate group (*n* = 35)	Loteprednol group (*n* = 27)	*P* value
Postoperative diagnosis, n (%)			0.691
Leukoma	12 (34.29)	6 (22.22)	
Cornea dystrophy	4 (11.43)	2 (7.41)	
Corneal degeneration	1 (2.86)	2 (7.41)	
Keratoconus	3 (8.57)	1 (3.70)	
Infectious corneal ulcer	12 (34.29)	13 (48.15)	
Infective corneal perforation	3 (8.57)	3 (11.11)	

Data were analyzed by χ^2^ test

### Risk factors for corticosteroid-induced ocular hypertension

No significant differences in the risk factors for corticosteroid-induced ocular hypertension, including aphakia, pseudophakia, cataracts and lenses, were observed after surgery between the two groups (*P* = 0.434) (Table 6).

**Table 6:** Risk factors of corticosteroid-induced ocular hypertension

Time	Prednisolone acetate group (*n* = 35)	Loteprednol group (*n* = 27)	*P* value
Lens status after transplantation, n (%)			0.434
Aphakia, n (%)	1 (2.86)	1 (3.70)	
Pseudophakia, n (%)	0	1 (3.70)	
Cataract, n (%)	0	1 (3.70)	
Lens, n (%)	34 (97.14)	24 (88.90)	

Data were analyzed by χ^2^ test

### Postoperative BCVA

As shown in Table 7, there were no significant differences in the preoperative average BCVA and average BCVA at 1, 3, 6 and 12 months postoperatively between the two groups. The average BCVA at 6 months postoperatively in the loteprednol group was significantly lower than that in the prednisolone acetate group (*P* = 0.046).

**Table 7:** Preoperative and postoperative mean BCVA between the two groups

Time	Prednisolone acetate group (*n* = 35)	Loteprednol group (*n* = 27)	*z* value	*P* value
Preoperative	2.7(1.3,3.0)	2.7(1.0,3.0)	-0.628	0.530
Postoperative 1 w	2.2(1.3,2.7)	2.4(1.0,3.0)	-0.666	0.506
Postoperative 1 m	1.3(1.0,2.2)	1.0(0.8,2.0)	-1.160	0.246
Postoperative 3 m	1.0(0.8,1.3)	1.0(0.9,2.0)	-0.215	0.830
Postoperative 6 m	1.1(0.8,2.0)	1.0(0.6,1.1)	-1.992	0.046
Postoperative 12 m	0.9(0.7,1.0)	0.9(0.5,1.3)	-0.584	0.559

*BCVA *best corrected visual acuity

Data were analyzed by Mann-Whitney U test

### Postoperative graft rejection rate and cataract rate

As shown in Table 8, there were no significant differences between the postoperative graft rejection rates in the two groups (*P* > 0.05). The postoperative cataract rate was 0 in the prednisolone acetate group and 1 (3.70 %) in the loteprednol group, and there were no significant differences between the two groups (*P* = 0.251).

**Table 8:** Postoperative follow-up graft rejection rate in the PKP group

	Prednisolone acetate group (*n* = 35)	Loteprednol group (*n* = 27)	*P* value
Graft rejection rate, n (%)	6 (17.14)	6 (22.22)	0.616

Data were analyzed by χ^2^ test

## Discussion

In this study, the effects of 1 % prednisolone acetate eye drops and 0.5 % loteprednol eye drops on IOP, vision and immunological rejection after PKP or LKP were retrospectively compared. 0.5 % loteprednol suspension eye drops raised the IOP less than 1 % prednisolone acetate eye drops at 6 months postoperatively, and similar vision loss and incidence of postoperative rejection were observed in both groups. Thus, 0.5 % loteprednol can be considered for long-term use after corneal transplantation.

Xie et al. [23] found that the incidence of secondary glaucoma after PKP was 5.3 %, of which steroid-induced glaucoma accounted for 45 %, suggesting that steroid-induced glaucoma accounts for a large proportion of secondary glaucoma after corneal transplantation. Fan et al. [24] found that the incidence of steroid-induced glaucoma was 35 % in 48 patients (57 eyes) who underwent PKP. Similarly, in our study, a total of 62 out of 234 eyes (26.5 %) developed corticosteroid-induced ocular hypertension after PKP or LKP. Akash [6] et al. conducted a PKP study on 596 eyes and found 96 eyes with corticosteroid-induced ocular hypertension, with an incidence of 16.9 %. The differences among the corticosteroid-induced ocular hypertension rates reported in different studies may be related to the tolerance, sensitivity, and primary disease of different populations. Francois [25] found that corticosteroid-induced ocular hypertension rarely occurs within 2 weeks after topical hormone treatment; however, the risk for corticosteroid-induced ocular hypertension cannot be ruled out even if it does not occur within 6 weeks. In our study, steroid-induced glaucoma was observed in 3 eyes (8.6 %), 10 eyes (28.6 %), 20 eyes (57.1 %) and 2 eyes (5.7 %) at 1, 3, 6 and 12 months postoperatively, respectively, in the prednisolone acetate group. Steroid-induced glaucoma was observed in 2 eyes (7.4 %), 16 eyes (59.3 %), 6 eyes (22.2 %) and 3 eyes (11.1 %) at 1, 3, 6 and 12 months postoperatively, respectively, in the loteprednol group. There was a high incidence of steroid-induced glaucoma 3–6 months postoperatively after corneal transplantation, which was consistent with previous research [26].

Thirty-three eyes (94.3 %) in the prednisolone acetate group and 26 eyes (96.3 %) in the loteprednol group had an IOP < 30 mmHg, which was consistent with the study of Xie Lixin that indicated that the typical IOP observed with steroid-induced glaucoma after corneal transplantation is < 30 mmHg [23].

Secondary glaucoma after corneal transplantation is recognized as an important factor that causes vision loss and even optic nerve atrophy. In this study, there were no significant differences in the preoperative average BCVA and average BCVA at 1, 3, 6 and 12 months postoperatively between the two groups. The average BCVA at 6 months postoperatively in the prednisolone group was significantly higher than that in the loteprednol group (*P* < 0.05). We speculated that this is because of the high incidence of immune rejection after corneal transplantation at 3–6 months, and the frequency of eye drop use at this stage is higher than that after 6 months. In addition, this finding may be related to individual patient tolerance to hormones.

Although corneal transplantation is the most successful organ transplantation procedure, postoperative rejection is still very common. Among the causes of postoperative rejection, immunological rejection is significant and causes transplantation failure in 27-34 % of patients [27]. Glucocorticoid eye drops are the main drugs used for the treatment of corneal graft rejection. According to previous studies, the incidence of rejection after PKP is 2.3-68 % [12, 28], and poor compliance after corneal transplantation and unauthorized discontinuation or reduction in the dosage of glucocorticoid eye drops are important causes of immunological rejection [29]. In this study, there were no significant differences in immunological rejection rates between the prednisolone group (6 eyes, 17.14 %) and the loteprednol group (6 eyes, 22.22 %).

Hormone-related high IOP levels after corneal transplantation mostly occurs at 3–6 months, and most patients have an IOP less than 30 mmHg. By reducing the frequency of eye drops and appropriately increasing the use of anti-ocular pressure drugs, hormone-related high IOP levels can be reversed. Both 0.5 % loteprednol suspension eye drops and 1 % prednisolone acetate eye drops raised the IOP, the incidence of high IOP and visual acuity at 6 months of follow-up, however the differences were statistically significant; the degree of IOP rise was within the range of mild to moderate, and there was no statistically significant difference between the two groups in terms of postoperative rejection.

The small sample size was a limitation of our study. In addition, the significantly reduced increase in the IOP observed with 0.5 % loteprednol suspension eye drops compared with 1 % prednisolone acetate eye drops disappeared at 12 months postoperatively. Therefore, a large randomized controlled study should be performed in the future to further evaluate the findings of this study.

## Conclusions

Treatment with 0.5 % loteprednol suspension dye drops reduced the increase in the IOP 6 months postoperatively compared with the IOP increase induced by 1 % prednisolone acetate eye drops; vision loss and incidence of postoperative rejection were not different between the two eye drop treatments. Thus, 0.5 % loteprednol suspension eye drops may be considered for long-term use after corneal transplantation.

## Data Availability

The datasets used during the current study are available from the corresponding author on reasonable request.

## Abbreviations

PKP: Penetrating keratoplasty
LKP: Lamellar keratoplasty
BCVA: Best corrected visual acuity
PK: Penetrating keratoplasty
LK: Lamellar keratoplasty
EK: Endothelial keratoplasty
DSEK: Descemets stripping endothelial keratoplasty
DMEK: Descemets membrane endothelial keratoplasty
IOP: Intraocular pressure
